# Butyl Rubber Nanocomposites with Monolayer MoS_2_ Additives: Structural Characteristics, Enhanced Mechanical, and Gas Barrier Properties

**DOI:** 10.3390/polym10030238

**Published:** 2018-02-27

**Authors:** Chi-Yang Tsai, Shuian-Yin Lin, Hsieh-Chih Tsai

**Affiliations:** 1 Graduate Institute of Applied Science and Technology, National Taiwan University of Science and Technology, Taipei 10607, Taiwan; charlie810903@gmail.com; 2 Industrial Technology Research Institute, Biomedical Technology and Device Research Laboratories, Hsinchu 31057, Taiwan; sylin@biip-mdcc.org

**Keywords:** layered structures, polymer-matrix composites, mechanical properties, gas barrier properties

## Abstract

Emerging two-dimensional (2D) materialsm, such as molybdenum disulfide (MoS_2_), offer opportunities to tailor the mechanical and gas barrier properties of polymeric materials. In this study, MoS_2_ was exfoliated to monolayers by modification with ethanethiol and nonanethiol. The thicknesses of resulting MoS_2_ monolayers were 0.7 nm for MoS_2_-ethanethiol and 1.1 nm for MoS_2_-nonanethiol. MoS_2_ monolayers were added to chlorobutyl rubber to prepare MoS_2_-butyl rubber nanocomposites at concentrations of 0.5, 1, 3, and 5 phr. The tensile stress showed a maximum enhancement of about 30.7% for MoS_2_-ethanethiol-butyl rubber and 34.8% for MoS_2_-nonanethiol-butyl rubber when compared to pure chlorobutyl rubber. In addition, the gas barrier properties were increased by 53.5% in MoS_2_-ethanethiol-butyl rubber and 49.6% in MoS_2_-nonanethiol-butyl rubber. MoS_2_ nanosheets thus enhanced the mechanical and gas barrier properties of chlorobutyl rubber. The nanocomposites that are presented here may be used to manufacture pharmaceutical stoppers with high mechanical and gas barrier properties.

## 1. Introduction

Since the discovery of graphene, two-dimensional inorganic materials, such as MoS_2_, have attracted great attention. MoS_2_ has a structure similar to that of graphite; two layers of sulfur and one layer of molybdenum atoms in a sandwiched structure make up its hexagonal crystal lattice structure. MoS_2_ is unreactive, unaffected by both acids and oxygen, and has a low coefficient of friction due to weak van der Waals interactions between the layers. As such, it is widely used as a dry lubricant. In addition, MoS_2_ can be exfoliated into nanolayers without the need for complex methods. MoS_2_ nanosheets have previously been utilized in transistors [1], biomaterials [2], and nanocomposites [3], and can also be added to polymers as a filler material; because of the high band gap of MoS_2_, the electronic properties of the polymer matrices are not changed. A common reason to add fillers to polymers is to improve their mechanical properties. For example, polymer chains can interact with the nanosheet surfaces, resulting in reinforcements in all directions from the nanosheets. For the latter, it is important to fully exfoliate the two-dimensional inorganic materials to increase the surface area [4].

Many studies have reported the use of nanoscale fillers such as clay, reduced graphene oxide, and MoS_2_ to improve the mechanical and gas barrier properties of polymer materials for a variety of applications. For example, the optimal mechanical or barrier properties were observed for exfoliated or intercalated polymer/clay nanocomposites, but using a high clay content of 5–10 wt % [5,6,7]. Clay is difficult to exfoliate due to the many cations in the spacing between the layers of the material. In addition, clay is hydrophilic and cannot disperse well in hydrophobic polymers. However, quaternary ammonium cation salt can usually act as modifiers to enable exfoliation and dispersion of clay molecules in polymer matrices [8]. Graphene consists of two-dimensional sheets of sp^2^-bonded carbon with a high specific surface area. Graphene-based nanocomposites play an important role because of their favorable mechanical, electrical, and barrier properties. Their barrier properties, for example, are much better than those of clay nanofillers [9,10,11]. Some applications require improvements in the mechanical properties and thermal stability of a polymer matrix, while maintaining the polymer’s electrical insulation properties. Graphene, as a highly conductive material, does not appear to be a good filler material choice for such applications. In addition, fillers have to be uniformly dispersed in a polymer matrix. However, exfoliation of graphene is still unpractical, with the most common method involving the treatment of graphite with strong oxidizers to obtain exfoliated graphene oxide. MoS_2_ exfoliation into nanosheets, on the other hand, can be achieved in a one-step, simple method at low MoS_2_ loading rates, and is thus more economical. MoS_2_ nanosheets are therefore an excellent alternative to clay and graphene-based materials for enhancing the properties of polymer matrices. MoS_2_ has been reported as filler to manufacture photo-mechanical response material [12], gas selective membranes [13], and supercapacitor [14].

Due to weak van der Waals interactions between the layers of bulk MoS_2_, MoS_2_ nanosheets can easily be prepared by ultrasonication. A common method for the exfoliation of MoS_2_ involves the use of lithium ions to intercalate the MoS_2_ nanosheets. However, it is hard to disperse MoS_2_ nanosheets in nonpolar polymers without modifying their surfaces with organic ligands. In previous works, ultrasonicating bulk MoS_2_ powder produced a number of sulfur vacancies on the surface of a MoS_2_ nanosheet, which were reported to act as targets for surface modification [15,16]. Here, thiol compounds were selected as modifiers of MoS_2_ nanosheets to increase the affinity between the nanosheets and polymer matrix. With a greater degree of MoS_2_ nanosheet dispersion, a greater degree of reinforcement would be expected in the nanocomposite.

Chlorobutyl rubber is often used in tires, gas masks, and chemical agent packaging because of its good mechanical and gas barrier properties. Unlike conventional butyl rubber, with a lack of double bond on the backbone of polymer chain, the vulcanization of chorobutyl rubber is more efficiently. The aim of this study was to enhance the mechanical and gas barrier properties of chemical agent packaging materials, which require enhanced gas barrier properties for the storage of chemical agents. For this reason, chlorobutyl rubber with added MoS_2_ was studied as a suitable material. Exfoliated MoS_2_ nanosheets surface-modified by ethanethiol and nonanethiol to enhance their affinity to polymers were expected to disperse well in chlorobutyl rubber and result in improved mechanical and gas barrier properties. Herein, the effects of ethanethiol- and nonanethiol-modified MoS_2_ nanosheets are compared for various MoS_2_ concentrations.

## 2. Materials and Methods

### 2.1. Materials

Chlorobutyl rubber (Mooney viscosity [ML_1+8_ 100 °C]: ~41–49) was obtain from ExxonMobil Chemical (Houston, TX, USA); MoS_2_ from Alfa Aesar (Haverhill, MA, USA); hexane from Fisher Chemical (Hampton, NH, USA); ethanethiol from Sigma-Aldrich (St. Louis, MO, USA); nonanethiol from Acros (Hampton, NH, USA); ethylenethiourea(2-mercaptoimidazoline) from Kawaguchi Chemical Industry (Kawaguchi, Japan); and silicon dioxide (TOKUSIL 255, with surface area BET 177 m^2^/g) was obtained from OSC Group (Miaoli, Taiwan).

### 2.2. Exfoliation of MoS_2_

For the exfoliation of MoS_2_, 400 mg MoS_2_ powder and 20 mL hexane were mixed in 20-mL vials and ethanethiol and nonanethiol were added to each vial. Ultrasonication to exfoliate MoS_2_ was applied in a bath for 24 h. After ultrasonication, the contents of the vials were allowed to settle, and exfoliated MoS_2_ was obtained as the suspension.

### 2.3. Preparation of MoS_2_-butyl Rubber Nanocomposites

MoS_2_-butyl rubber nanocomposites were prepared with various MoS_2_ concentrations 0.5, 1, 3, and 5 parts per hundreds of rubber (phr). The previously obtained MoS_2_ nanosheets were mixed with chlorobutyl rubber and were dissolved in hexane under mechanical stirring for 1 h to achieve a homogenous mixture. The hexane was then evaporated and the samples thus obtained were dried at 100 °C in a vacuum oven for 12 h to completely remove the solvent. The samples were compounded by two-roll-mill with 20 phr silicon dioxide as a widely used filler for rubber to improve the wear resistance and also acts as a reinforcing agent and using 0.5 phr ethylenethiourea(2-mercaptoimidazoline) as the curing reagent. After compression molding at 185 °C at a pressure of 50 kgf/cm^2^ for 10 min, MoS_2_-butyl rubber nanocomposite samples with dimensions of 15 cm × 15 cm and a 1-mm thickness were obtained.

### 2.4. Characterization

The morphologies of the MoS_2_ nanosheets modified by ethanethiol and nonanethiol were observed using a Tecnai™ G2 F-20 (Philips, Amsterdam, Netherlands) transmission electron microscope (TEM). Raman spectra and Raman maps were obtained using an NRS5100 (JASCO, Tokyo, Japan) spectrometer. Cross-sectional images were obtained using a JSM-6500F (JEOL, Tokyo, Japan) scanning electron microscope (SEM); and, composite samples were cooled in liquid nitrogen and cut by a scalpel to prepare the samples for backscattered electron (BSE) imaging. Atomic force microscopy (AFM) was performed using a NX10 system (Park, Suwon, Korea). X-ray diffraction (XRD) was performed using a D8 SSS (Bruker, Billerica, MA, USA). UV-Vis spectra were obtained using a V-730 spectrometer (JASCO, Tokyo, Japan). Dynamic mechanical analysis was performed using a Q800 (TA Instruments, New Castle, DE, USA), while stress-strain curves were measured using a TS-2000 with a crosshead speed of 500 mm/min. The oxygen transmission rates were measured according to the ASTM D3985 standard using the OX-TRAN 2/61 (Mocon Inc., Minneapolis, MN, USA) at 23 °C and a relative humidity of 0%; film specimens of 5 cm in diameter and 1 mm in thickness were fixed between two chambers, and oxygen filled the upper chamber while nitrogen filled the lower chamber.

## 3. Results and Discussion

### 3.1. Exfoliation of MoS_2_

**Scheme 1:**
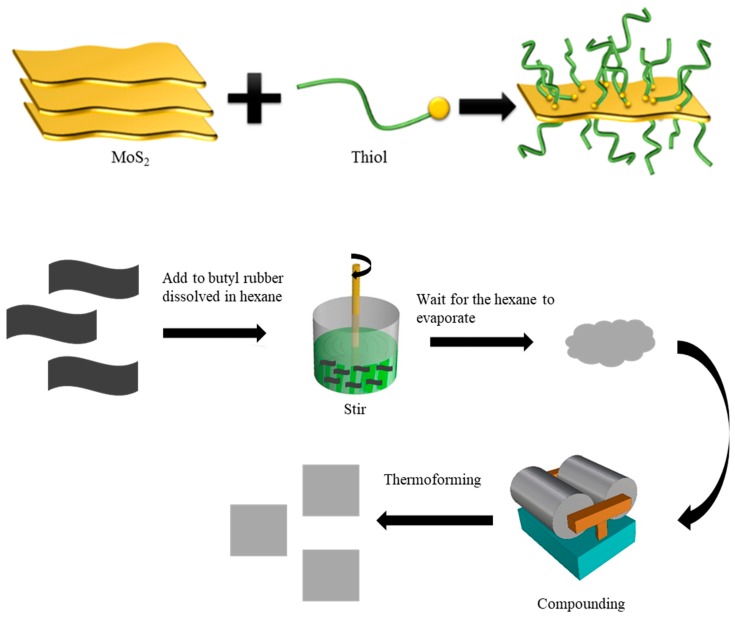
Schematic illustration for exfoliation modification of MoS_2_ and corresponding production of chlorobutyl rubber-based nanocomposites.

Scheme 1 outlines the overall procedure for the preparation of the MoS_2_ nanosheets and the production of MoS_2_-butyl rubber nanocomposites. The exfoliation of MoS_2_ was achieved by bath ultrasonication of bulk MoS_2_ powder in hexane. It has previously been reported that this exfoliation process can produce a number of structural defects, such as S vacancy defects [17,18]. Then, MoS_2_ nanosheets can be modified with thiol ligands. Ethanethiol and nonanethiol were used as the surface modifiers in this study. The carbon chains of these two thiols were hypothesized to modify the surface of MoS_2_ to enhance its compatibility with chlorobutyl rubber. The organic modification of the surface and robust nature of the modifiers ensured good dispersion and a dramatically enhanced properties of the polymer materials.

**Figure 1:**
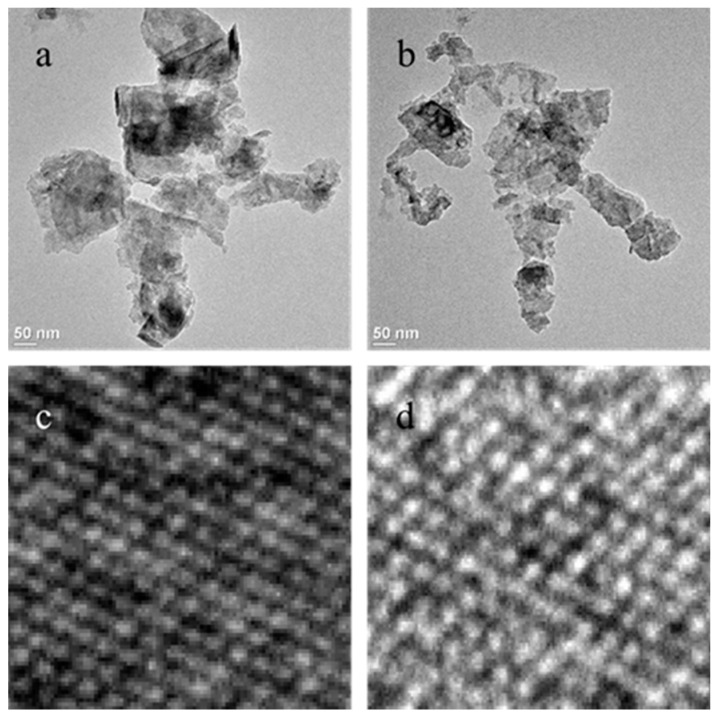
Transmission electron microscope (TEM) images of MoS_2_ nanosheets. (**a**,**c**) Low- and high-resolution images of MoS_2_-ethanethiol; (**b**,**d**) low- and high-resolution images of MoS_2_-nonanethiol.

**Figure 2:**
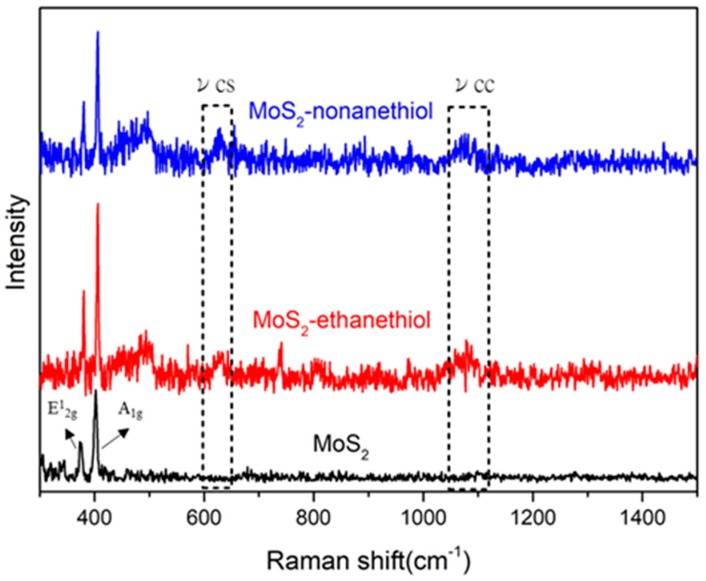
Raman spectra of MoS_2_ and thiol-modified MoS_2_.

The morphologies of MoS_2_ nanosheets modified by ethanethiol and nonanethiol are presented in TEM images (Figure 1a,b). The hexagonal structure of MoS_2_ modified by ethanethiol and nonanethiol was clearly visible in high-resolution TEM images (Figure 1c,d). It can be inferred that these MoS_2_ nanosheets were either several layers thick or monolayers, because the hexagonal lattice structure of MoS_2_ was visible. The latter indicates that the crystal structures of MoS_2_-ethanethiol and MoS_2_-nonanethiol were retained during ultrasonication [19]. Raman spectra were used to confirm the modification of the MoS_2_ nanosheet surfaces by ethanethiol and nonanethiol (Figure 2). Peaks were seen at ~380 cm^−1^ (E^1^_2g_, in-plane vibrations) and ~410 cm^−1^ (A_1g_, out-of-plane vibrations), characteristic of the MoS_2_ trigonal structure. Peaks at ~680 and ~1100 cm^−1^, which indicate carbon-sulfur (*ν*_cs_) [20] and carbon-carbon bonds (*ν*_cc_) [21], respectively, were noted for the modified MoS_2_. These results indicate that the surface of MoS_2_ was successfully modified by ethanethiol and nonanethiol.

**Figure 3:**
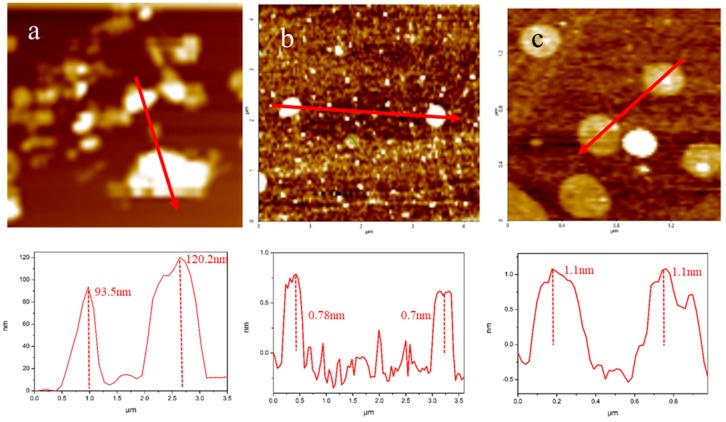
Atomic force microscope (AFM) images of: (**a**) bulk MoS_2_, (**b**) MoS_2_-ethanethiol, and (**c**) MoS_2_-nonanethiol.

**Figure 4:**
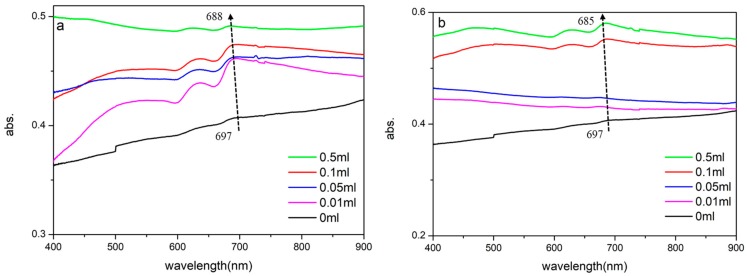
UV-Vis spectra: (**a**) MoS_2_-ethanethiol and (**b**) MoS_2_-nonanethiol.

The thicknesses of the exfoliated nanosheets were monitored through AFM examination of the exfoliated samples. The thickness of bulk MoS_2_ was ~90–120 nm (Figure 3a), while that of MoS_2_-ethanethiol was ~0.7 nm (Figure 3b) and that of MoS_2_-nonanethiol was ~1.1 nm (Figure 3c), values that correspond to that of ~0.65 nm in previous reports on the thickness of MoS_2_ monolayers [1]. The thicknesses obtained here being greater than the typical thickness of a single-layer MoS_2_ sheet may be attributed to thiol conjugation on the surface of MoS_2_ [22]. Blue-shifts of UV-Vis spectra are dependent on changes in the band gap energy, which can be obtained from the wavelengths in UV-Vis spectra from the following equation:

Band gap energy (*E*) = (*hc*)/λ (1)

where *hc* is Planck’s constant and λ is the wavelength. Bulk MoS_2_ is an indirect semiconductor with a band gap of ~1.2 eV, which increases to ~1.8 and ~1.9 eV for monolayers of MoS_2_ [23,24]. To obtain the optimum parameters for exfoliation, the number of MoS_2_ nanosheet layers was measured for various concentrations of ethanethiol and nonanethiol by UV-Vis spectra (Figure 4). The MoS_2_-ethanethiol sample in Figure 4a shows a blue-shift from 697 to 688 nm. The latter wavelength of 688 nm corresponds to a band gap value of 1.80 eV. For MoS_2_-nonanethiol in Figure 4b, a blue-shift from 697 to 685 nm can be observed. The latter wavelength of 685 nm corresponds to a band gap value of 1.81 eV. The conditions to exfoliate MoS_2_ into monolayer involved the addition of 0.5 mL of either ethanethiol or nonanethiol with 400 mg bulk MoS_2_ powder into 20 mL hexane. The exfoliation efficiency for MoS_2_ that was treated with nonanethiol was greater than that of MoS_2_ treated with ethanethiol.

### 3.2. Characterization of MoS_2_-butyl rubber Nanocomposites

XRD was performed to characterize the obtained layered-structure materials and partially evaluate the dispersion state of layered nanofillers in the polymer composites. XRD scans of the polymer nanocomposites showed a nanofiller peak and a shift to a lower 2θ or larger d-spacing value when compared to bulk MoS_2_. The peak shift indicates an expansion of the d-spacing of MoS_2_ nanosheets; it was inferred that polymer chains had been intercalated in the MoS_2_ nanosheets. For completely exfoliated layered nanofillers, no XRD peaks were expected for the nanocomposites, since they should not show regular spacing of the sheets [25].

**Figure 5:**
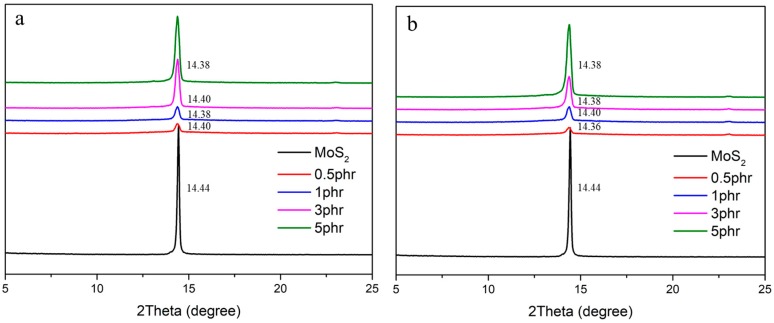
X-ray diffraction (XRD) patterns of MoS_2_-butyl rubber nanocomposites: (**a**) MoS_2_-ethanethiol-butyl rubber and (**b**) MoS_2_-nonanethiol-butyl rubber.

The XRD patterns (Figure 5) of the MoS_2_-butyl rubber nanocomposites confirm the intercalation of chlorobutyl rubber in the MoS_2_ nanosheet interlayers by showing a decrease in 2θ value as the concentration of MoS_2_ increased. The (002) peak of pure MoS_2_ was at 2θ = 14.44°, corresponding to a d-spacing value of 0.3088 nm. After adding MoS_2_ to chlorobutyl rubber, the 2θ peak of the (002) plane shifted to lower angles, associated with intercalation in nanocomposites. For MoS_2_-ethanethiol-butyl rubber, the peak at 2θ = 14.44° (*d* = 0.3088 nm) for 0 phr shifted to 2θ = 14.40° (*d* = 0.3097 nm), and 2θ = 14.38° (*d* = 0.3102 nm) for the samples with 3 and 5 phr MoS_2_, respectively. For MoS_2_-nonanethiol-butyl rubber, the peak was at 2θ = 14.36° for the 0.5-phr sample, which indicates that the d-spacing of MoS_2_ increased when MoS_2_ nanosheets were inserted into the chlorobutyl rubber chains. The latter illustrates that, between the exfoliation and intercalation, the nanocomposites can be driven toward full exfoliation by decreasing the content of MoS_2_ nanosheets. The greater shift at low concentrations indicates that nonanethiol is a more suitable modifier for MoS_2_ exfoliation than ethanethiol.

**Figure 6:**
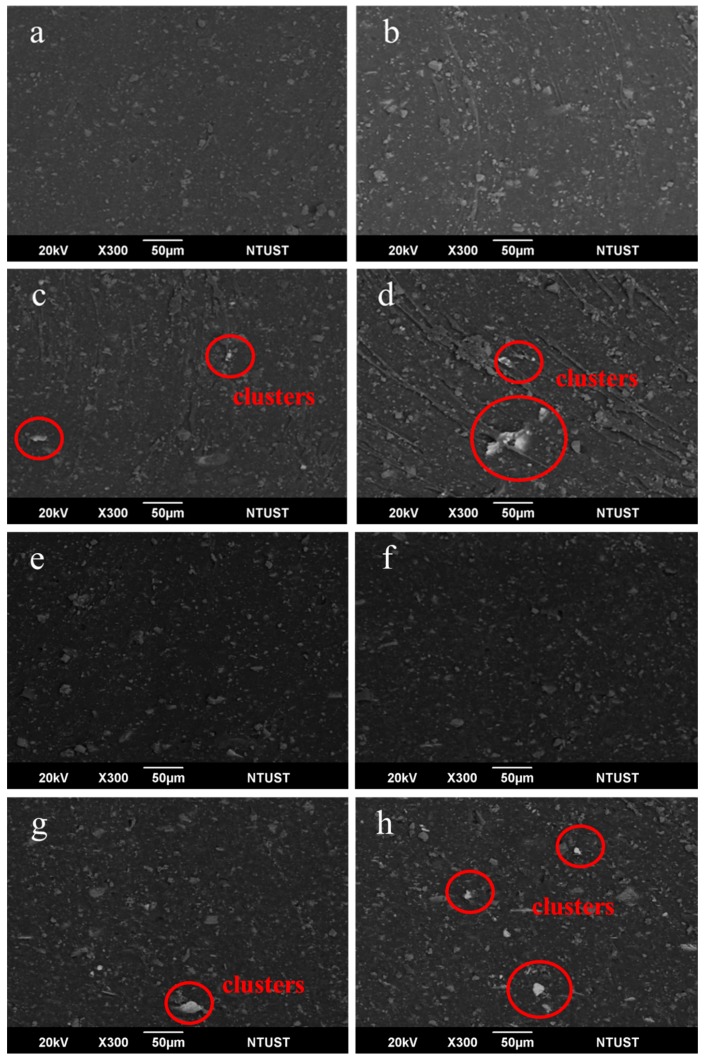
Scanning electron microscope backscattered electrons (SEM-BSE) cross-sectional images for MoS_2_-butyl rubber with different concentrations of MoS_2_ with either ethanethiol or nonanethiol: (**a**) 0.5 phr, ethanethiol; (**b**) 1 phr, ethanethiol; (**c**) 3 phr, ethanethiol; (**d**) 5 phr, ethanethiol; (**e**) 0.5 phr, nonanethiol; (**f**) 1 phr, nonanethiol; (**g**) 3 phr, nonanethiol; and, (**h**) 5 phr, nonanethiol.

The SEM-BSE images (Figure 6) of MoS_2_-butyl rubber nanocomposite cross-sections demonstrate the dispersion of MoS_2_ nanosheets in chlorobutyl rubber obtained at different concentrations. These micrographs confirm that, at higher concentrations, i.e., 3 and 5 phr, big clusters of agglomerated ethanethiol- and nonanethiol-modified MoS_2_ were present. At lower concentrations, i.e., 0.5 and 1 phr, on the other hand, MoS_2_ was homogeneously dispersed in chlorobutyl rubber.

**Figure 7:**
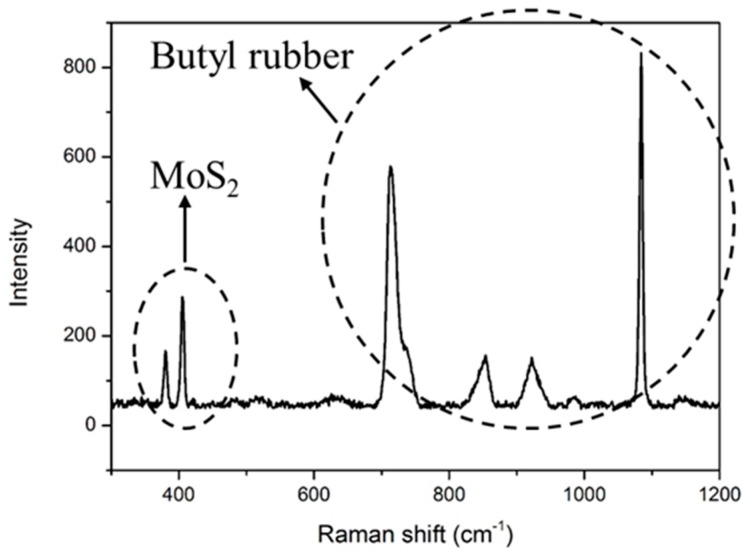
Typical Raman peaks for MoS_2_-butyl nanocomposites.

**Figure 8:**
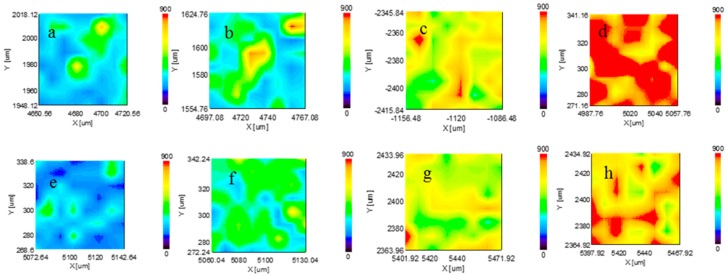
Raman mapping images for MoS_2_-butyl rubber with different concentrations of MoS_2_ with either ethanethiol or nonanethiol: (**a**) 0.5 phr, ethanethiol; (**b**) 1 phr, ethanethiol; (**c**) 3 phr, ethanethiol; (**d**) 5 phr, ethanethiol; (**e**) 0.5 phr, nonanethiol; (**f**) 1 phr, nonanethiol; (**g**) 3 phr, nonanethiol; and, (**h**) 5 phr, nonanethiol.

The typical Raman peaks for MoS2-butyl rubber nanocomposites are shown in Figure 7. The peaks at ~380 and ~410 cm^−1^ correspond to MoS2, while the peaks at ~720, ~820, ~910, and ~1080 cm^−1^ correspond to chlorobutyl rubber. Raman mapping (Figure 8) was used to further confirm the dispersion state of MoS_2_ nanosheets at different MoS_2_ concentrations. Figure 8 shows the intensity maps of the A_1g_ peak (~410 cm^−1^) of MoS_2_ for nanocomposites with different concentrations of modified MoS_2_ nanosheets. The Raman mapping images correspond well with the SEM-BSE images (Figure 6). At low concentrations of MoS_2_ nanosheets, their distribution was uniform, which implies homogeneous dispersion in chlorobutyl rubber. As the MoS_2_ loading increased, however, agglomeration and clustering behavior of the MoS_2_ was visible, illustrating poor dispersion. Nonetheless, due to their conjugation with ethanethiol or nonanethiol, MoS_2_ nanosheets could disperse homogeneously in chlorobutyl rubber at low concentrations. As shown in Figure 6, MoS_2_-nonanethiol-butyl rubber had a more uniform appearance than MoS_2_-ethanethiol-butyl rubber; at 5 phr MoS_2_, in particular, the clustering for MoS_2_-ethanethiol-butyl rubber was more pronounced than for MoS_2_-nonanethiol-butyl rubber.

### 3.3. Tensile Properties of MoS_2_-butyl Rubber Nanocomposites

**Figure 9:**
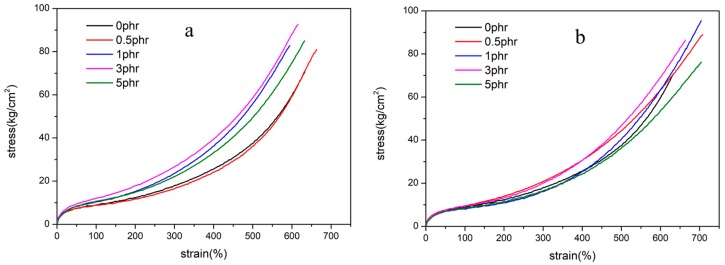
Stress-strain curves: (**a**) MoS_2_-ethanethiol-butyl and (**b**) MoS_2_-nonanethiol-butyl rubber.

The stress-strain curves (Figure 9) for neat chlorobutyl rubber and MoS_2_-butyl rubber nanocomposites show that the tensile strength of the chlorobutyl rubber matrix increased upon MoS_2_ nanosheet loading. Furthermore, the elongation at break of MoS_2_-nonanethiol-butyl rubber was about 14.4% higher than that of MoS_2_-ethanethiol-butyl rubber. The maximum increase in tensile strength for MoS_2_-ethanethiol-butyl rubber was about 30.7% for a MoS_2_ content of 3 phr. In MoS_2_-nonanethiol-butyl rubber, likewise, the tensile strength was increased by about 34.8% for 1 phr MoS_2_ as compared to that of the control sample. Therefore, the maximum increase in tensile strength was obtained for MoS_2_-nonanethiol-butyl rubber instead of MoS_2_-ethanethiol-butyl rubber. The significant increase in tensile strength reached a peak at a loading of 3 phr for MoS_2_-ethanethiol-butyl rubber and of 1 phr for MoS_2_-nonanethiol-butyl rubber. At higher MoS_2_ nanosheet contents, the tensile strength decreased again. The latter observations may be ascribed to the aggregation of MoS_2_ nanosheets in the chlorobutyl rubber matrix, which is known to cause a decrease in tensile strength for rubber [26]. It is obvious from these results that MoS_2_ nanosheets can significantly improve the strength of chlorobutyl rubber, possibly due to the high strength of MoS_2_ nanosheets, better interactions between MoS_2_ nanosheets and the polymer matrix, and/or a more uniform dispersion of MoS_2_ nanosheets in the chlorobutyl rubber matrix due to abundant thiol groups on the MoS_2_ nanosheet surfaces.

### 3.4. Dynamic Mechanical Analysis of MoS_2_-butyl Rubber Nanocomposites

**Figure 10:**
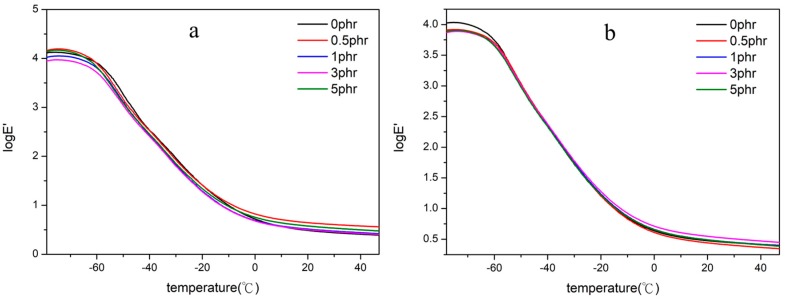
Storage modulus measurements: (**a**) MoS_2_-ethanethiol-butyl and (**b**) MoS_2_-nonanethiol-butyl rubber.

For MoS_2_-ethanethiol-butyl rubber, the storage modulus (Figure 10a) is a measure of its stiffness and the elastic of material that means the ability to recover pristine shape, and it a little increased for all the MoS_2_-butyl rubber nanocomposites in rubbery region compared to pure chlorobutyl rubber but no significant increment in glassy region. In rubbery region, the nanocomposite containing 0.5 phr MoS_2_ nanosheets exhibited the highest modulus value. MoS_2_-nonanethiol-butyl rubber also showed an increase in the storage modulus (Figure 10b), with an increase in the content of MoS_2_ nanosheets, except for 0.5 phr, and reached the highest modulus value for 3 phr. These results indicate that MoS_2_ nanosheet incorporation into chlorobutyl rubber remarkably enhanced stiffness and had a significant reinforcing effect. This increase in storage modulus results from the intercalation of MoS_2_ nanosheets in chlorobutyl rubber and strong interactions between the chlorobutyl rubber polymer chain and MoS_2_ nanosheets. The mobility of the polymer chains in rubbery region was thus retarded by the MoS_2_ nanosheets, resulting in the higher storage modulus.

**Figure 11:**
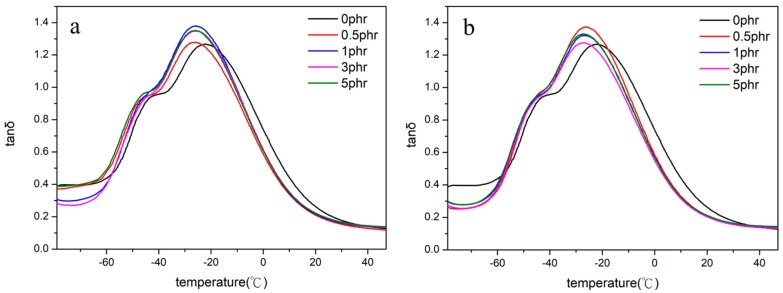
tan(*δ*) Measurements: (**a**) MoS_2_-ethanethiol-butyl and (**b**) MoS_2_-nonanethiol-butyl rubber.

The tan(δ) values of MoS_2_-ethanethiol-butyl rubber are shown in Figure 10a. For all of the samples of MoS_2_-ethanethiol-butyl rubber, shifts to lower temperatures were observed when compared to the 0 phr sample. MoS_2_ intercalated in chlorobutyl rubber may act as a lubricant, which leads to lowering of the glass transition temperature [27]. The tan(δ) values of MoS_2_-nonanethiol-butyl rubber are shown in Figure 11b; similar shifts to lower temperatures can be seen, again indicating intercalation of MoS_2_ nanosheets in the chlorobutyl rubber. The barrier effect of the nano-flakes restricting the motion of the polymer chains in the nanocomposites can be ascribed to the MoS_2_ nanosheets.

### 3.5. Gas Barrier Properties of MoS_2_-butyl Rubber Nanocomposites

**Scheme 2:**
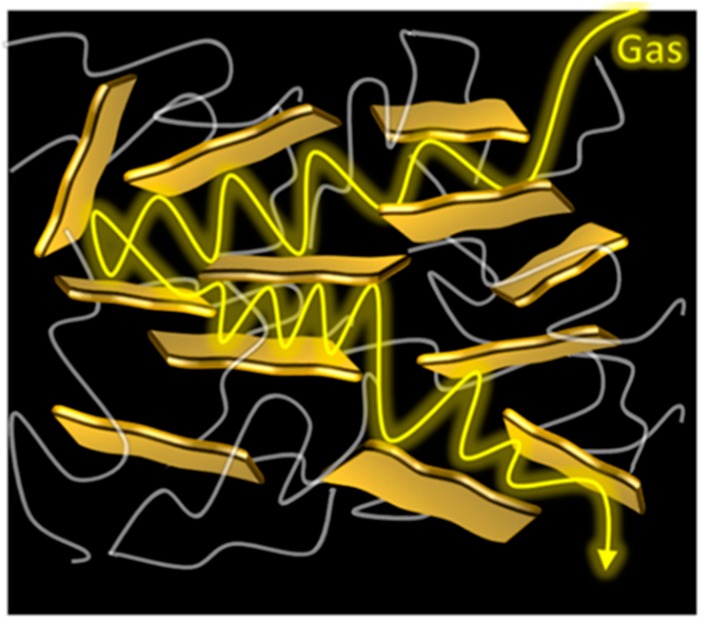
Barrier to permeation imposed by nanoparticles embedded in a polymeric matrix.

The barrier properties of polymers can be significantly altered by including sufficient inorganic platelets to alter the path of gas molecules (Scheme 2) [4]. The oxygen transmission rate (OTR) (Table 1) of each MoS_2_-butyl rubber nanocomposite was measured at 25 °C using the method outlined by ASTM D3985. When compared to that of pure chlorobutyl rubber, the OTR of MoS_2_-ethanethiol-butyl rubber decreased dramatically to 42.3 cc/m^2^-day at the MoS_2_ nanosheet concentration of 0.5 phr. The OTR of MoS_2_-nonanethiol-butyl rubber decreased to 47.2 cc/m^2^-day at 0.5 phr, and thereafter decreased slowly at higher concentrations. The barrier performance for all MoS_2_-butyl rubber nanocomposites could be improved markedly by the application of a small amount of organic-modified MoS_2_. Moreover, there was little difference between the gas barriers of MoS_2_-ethanethiol-butyl rubber and MoS_2_-nonanethiol-butyl rubber, since the surface areas of MoS_2_-ethanethiol and MoS_2_-nonanethiol nanosheets were too small to retard the pathway of gas molecules. There are two reasons behind the enhancement of the gas barrier properties of the MoS_2_-butyl rubber nanocomposites. First, MoS_2_ nanosheets form tortuous pathways in chlorobutyl rubber, which retard the progress of gas molecules through the composite. Secondly, the diffusion coefficient of the gas molecules decreases because MoS_2_ nanosheets strongly restrict the motion of the polymer chains [7].

## 4. Conclusions

In conclusion, we have demonstrated that MoS_2_ nanosheets are an excellent filler material to enhance the tensile properties of chlorobutyl rubber. Ethanethiol and nonanethiol played an important role in modifying the surface of MoS_2_ nanosheets. Using thiol modification of nanosheets helped to obtain MoS_2_ monolayers with a thickness of ~0.8–1 nm, a key feature of MoS_2_ nanosheets intercalated in chlorobutyl rubber. The obtained MoS_2_ nanosheets were dispersed homogeneously in chlorobutyl rubber due to the thiol ligands modifying MoS_2_ to enable greater affinity between MoS_2_ and chlorobutyl rubber. Due to the high stiffness of the MoS_2_ nanosheets, MoS_2_ improved the mechanical properties of chlorobutyl rubber in tensile test, but not significantly in storage modulus. On the other hand, the gas barrier was improved dramatically, although similarly for MoS_2_-ethanethiol- and MoS_2_-nonanethiol-butyl rubber. These results offer new opportunities utilizing nanocomposites of polymers and MoS_2_. Controlling the dimensions of MoS_2_ nanosheets remains a challenge. Therefore, improved techniques are necessary to produce MoS_2_ nanosheets of appropriate sizes, which can then achieve their full potential in polymer nanocomposites.

## Figures and Tables

**Table 1:** Oxygen transmission rates (OTRs) of MoS_2_-butyl rubber nanocomposites.

	MoS_2_-ethanethiol-butyl rubber (cc/m^2^-day)	MoS_2_-nonanethiol-butyl rubber (cc/m^2^-day)
0 phr	90.9	90.9
0.5 phr	42.3	47.2
1 phr	48.2	46.8
3 phr	44.6	45.8
5 phr	43.7	46.5
